# Management of tailgut cysts via three-dimensional-modeled Kraske procedure

**DOI:** 10.1590/1806-9282.20260883

**Published:** 2026-09-07

**Authors:** Milan Tesar, Ilker Sengul, Demet Sengul, Ivana Mrazkova, Lubomir Martinek, Anton Pelikan, Jan Hrubovcak

**Affiliations:** 1 University Hospital Ostrava, Department of Surgery – Ostrava, Czech Republic.; 2 University of Ostrava, Faculty of Medicine, Department of Surgical Studies – Ostrava, Czech Republic.; 3 Giresun University, Faculty of Medicine, Thyroidology Unit – Giresun, Turkey.; 4 Giresun University, Faculty of Medicine, Division of Endocrine Surgery – Giresun, Turkey.; 5 Giresun University, Faculty of Medicine, Department of General Surgery – Giresun, Turkey.; 6 Giresun University, Faculty of Medicine, Department of Pathology – Giresun, Turkey.; 7 Tomas Bata University, Faculty of Humanities, Department of Health Care Sciences – Zlin, Czech Republic.

The intricate landscape of the retrorectal space often harbors rare and complex pathologies that challenge the diagnostic acumen of even the most seasoned clinicians. Among these, the tailgut cyst (TGC), or retrorectal cystic hamartoma, stands as a testament to the complexities of human embryogenesis. Arising from the incomplete involution of the embryonic hindgut’s caudal extremity, these lesions are sequestered within the presacral potential space. While extant literature frequently highlights a marked female predominance—often cited at a ratio of 3:1 to 4:1—it is our contention that this demographic distribution may be partially skewed by the frequency of routine gynecological surveillance in women, whereas in men, these lesions may remain sequestered until advanced stages or symptomatic progression^1,2,3,4^.

The clinical presentation of TGCs is notoriously protean. Approximately half of the affected individuals remain asymptomatic, with the remaining cohort presenting with symptoms secondary to local mass effect, such as constipation or urinary dysfunction. However, the case of a 39-year-old male presenting with chronic pelvic discomfort and post-ejaculatory pain warrants specific academic attention. The latter symptom is particularly infrequent in the context of TGCs and underscores the potential for these lesions to mimic primary urological or sexual dysfunctions, thereby delaying the definitive diagnosis by months or even years. In the male population, such delays are not merely a matter of prolonged morbidity but are intrinsically linked to an elevated risk of malignant transformation—a complication reported with varying frequency in major series, involving transitions to adenocarcinomas, carcinoids, or more rarely, squamous cell carcinomas^3,5^. The diagnostic cornerstone for retrorectal masses remains magnetic resonance imaging (MRI), owing to its superior soft-tissue characterization and multiplanar capability. In the case at hand, MRI and computed tomography (CT) revealed a significant cystic mass measuring 51 mm×56 mm×42 mm. Yet, the anatomical constraints of the pelvic basin often limit the surgeon’s perspective during conventional imaging review.

As such, to transcend these limitations, we integrated preoperative three-dimensional (3D) modeling, utilizing 3D slicer software in order to create a tactile, high-fidelity replica of the patient’s pelvic anatomy. This approach facilitates a “pre-emptive” surgical exploration, allowing the operative team to delineate the precise interface between the cyst, the rectum, the prostate, and the sacral nerves. We posit that 3D modeling represents the next frontier in surgical precision for retrorectal pathology, transforming two-dimensional data into a comprehensive spatial roadmap. The selection of the surgical corridor is paramount. While anterior approaches are generally reserved for lesions superior to the S3 level, the posterior transsacral approach, or Kraske procedure, remains the gold standard for low-lying retrorectal cysts. The utilization of the jackknife position and the judicious resection of the distal coccyx provided optimal exposure for the complete extirpation of the lesion in our patient. The necessity of complete excision cannot be overstated; incomplete removal not only invites recurrence but also leaves the patient vulnerable to future malignancy within the residual epithelial lining. Intraoperative adjuncts, such as rectoscopy, serve as vital safety maneuvers to ensure the integrity of the rectal wall, particularly when the cyst is intimately adherent to the mesorectal fascia.

Histopathological confirmation is the final, definitive step in management. The identification of diverse epithelia—ranging from squamous to mucus-secreting columnar types—without skin adnexa is pathognomonic for TGC. The absence of atypia in this case was a favorable outcome; however, the literature serves as a sobering reminder of the aggressive potential of these embryological vestiges. Consequently, a rigorous follow-up regimen involving clinical assessment and periodic imaging is essential, as standardized surveillance protocols remain largely ill-defined^1,2,3,4,5,6,7^. Although rare, TGCs should be considered in the differential diagnosis of presacral and retrorectal masses, particularly in middle-aged women presenting with unexplained anorectal^8,9^ or pelvic symptoms. Early detection using CT, and preferably MRI, followed by appropriate surgical management, is essential to prevent complications and malignant transformation. It can be assumed that these cysts are underdiagnosed in men and may be more common than previously thought.

Managing a TGC is a form of “clinical archeology.” Much like an archeologist carefully unearths a relic of a bygone era, the surgeon must delicately extract a vestige of the patient’s own embryonic history that has remained buried and forgotten in the deep strata of the pelvis. This lesion is a “biological ghost,” a haunting reminder of a developmental process that failed to fully conclude, lingering in the shadows of the retrorectal space. The surgical application of 3D modeling serves as a “GPS for the anatomical wilderness.” Without it, the surgeon navigates a complex terrain of nerves and vessels based on traditional maps; with it, they possess a real-time, 3D guide that illuminates the safest path. The Kraske procedure itself acts as a “rear-entry vault key,” providing the specific access necessary to unlock the presacral space without disturbing the delicate “front-facing” structures of the abdomen. Ultimately, the successful excision of a TGC is the act of “closing an open congenital loop,” thereby completing the involution that should have occurred in the womb and restoring the patient‘s physiological harmony.

In conclusion, the management of TGCs requires a multidisciplinary synthesis of embryological knowledge, advanced radiological interpretation, and meticulous surgical execution. The integration of 3D modeling represents a significant leap forward in optimizing these parameters. Of note, we must remain vigilant in our diagnostic approach to pelvic pain in men, ensuring that these “hidden” congenital anomalies are identified and treated before they transition from benign curiosities to malignant threats.

## Data Availability

The datasets generated and/or analyzed during the current study are available from the corresponding author upon reasonable request.
